# Induction immunotherapy plus chemotherapy followed by definitive chemoradiation therapy in locally advanced esophageal squamous cell carcinoma: a propensity-score matched study

**DOI:** 10.1007/s00262-024-03649-x

**Published:** 2024-02-16

**Authors:** Hui-min Lian, Jia-liang Wu, Wei-jian Liufu, Tian-tian Yu, Shao-qing Niu, Yong Bao, Fang Peng

**Affiliations:** 1 Department of Radiation Oncology, The First Affiliated Hospital of Sun Yat-Sen University, 58 Zhongshan Road II, Guangzhou, 510080 Guangdong Province China; 2 Shenzhen Qianhai Taikang Hospital, Shenzhen, 518000 Guangdong Province China

**Keywords:** Esophageal cancer, Immunotherapy, Induction chemotherapy, Concurrent chemoradiotherapy, Unresectable esophageal squamous cell carcinoma

## Abstract

**Background:**

For patients with unresectable locally advanced esophageal squamous cell carcinoma (ESCC), concurrent chemoradiotherapy (CCRT) is the current standard treatment; however, the prognosis remains poor. Immunotherapy combined with chemotherapy has demonstrated improved survival outcomes in advanced ESCC. Nevertheless, there is a lack of reports on the role of induction immunotherapy plus chemotherapy prior to CCRT for unresectable locally advanced ESCC. Therefore, this study aimed to evaluate the efficacy and safety of induction immunotherapy plus chemotherapy followed by definitive chemoradiotherapy in patients with unresectable locally advanced ESCC.

**Methods:**

This study retrospectively collected clinical data of patients diagnosed with locally advanced ESCC who were treated with radical CCRT between 2017 and 2021 at our institution. The patients were divided into two groups: an induction immunotherapy plus chemotherapy group (induction IC group) or a CCRT group. To assess progression-free survival (PFS) and overall survival (OS), we employed the Kaplan–Meier method after conducting propensity score matching (PSM).

**Results:**

A total of 132 patients with unresectable locally advanced ESCC were included in this study, with 61 (45.26%) patients in the induction IC group and 71 (54.74%) patients in the CCRT group. With a median follow-up of 37.0 months, median PFS and OS were 25.2 and 39.2 months, respectively. The patients in the induction IC group exhibited a significant improvement in PFS and OS in comparison with those in the CCRT group (median PFS: not reached [NR] versus 15.9 months, hazard ratio [HR] 0.526 [95%CI 0.325–0.851], *P* = 0.0077; median OS: NR versus 25.2 months, HR 0.412 [95%CI 0.236–0.719], *P* = 0.0012). After PSM (50 pairs), both PFS and OS remained superior in the induction IC group compared to the CCRT group (HR 0.490 [95%CI 0.280–0.858], *P* = 0.011; HR 0.454 [95%CI 0.246–0.837], *P* = 0.0093), with 2-year PFS rates of 67.6 and 42.0%, and the 2-year OS rates of 74.6 and 52.0%, respectively. Multivariate analysis revealed that lower tumor stage, concurrent chemotherapy using double agents, and induction immunotherapy plus chemotherapy before CCRT were associated with better prognosis.

**Conclusions:**

Our results showed for the first time that induction immunotherapy plus chemotherapy followed by CCRT for unresectable locally advanced ESCC provided a survival benefit with manageable safety profile. More prospective clinical studies should be warranted.

## Introduction

Esophageal cancer (EC) ranks as the sixth leading cause of cancer-related death worldwide, resulting in approximately 5.4 million deaths annually [1, 2]. Substantial variations exist in the incidence, mortality, and histopathology of esophageal cancer across different geographic regions [3, 4]. China exhibits a high prevalence of EC with over 50% of new cases reported globally each year; more than 90% of these cases are attributed to squamous cell carcinoma [5, 6]. 

Surgery is the preferred treatment modality for patients with locally advanced EC [7]; however, at the time of diagnosis, approximately 50–60% of EC patients are ineligible for radical resection [8]. Concurrent chemoradiotherapy (CCRT) currently serves as the standard therapeutic approach for unresectable locally advanced esophageal squamous cell carcinoma (ESCC) [9–12]. Despite advancements in treating this population, a considerable number of patients eventually experience local recurrence or distant metastases, leading to a disappointing prognosis [4, 13–15]. Previous studies have attempted to optimize CCRT regimens in order to enhance patient survival; nevertheless, altering chemotherapy agents or increasing radiotherapy dosage have not yielded significant improvements in survival rates [11, 15, 16]. The efficacy of induction chemotherapy in improving survival outcomes for patients with unresectable locally advanced ESCC remains controversial based on existing research findings [17–20]. Therefore, it is imperative to identify a novel and effective regimen for managing unresectable locally advanced ESCC.

In recent years, the clinical application of immune checkpoint inhibitors (ICIs) has significantly improved the prognosis of various malignant tumors, including EC [21–25]. Multiple randomized clinical trials have consistently demonstrated that anti-programmed cell death-1 (PD-1) inhibitors, as first- or second-line agents, substantially enhance overall survival in patients with advanced EC [21–28]. The combination of anti-PD-1 immunotherapy and chemotherapy has emerged as the new standard first-line treatment for advanced EC [21, 22, 25, 29, 30]. Furthermore, several clinical studies have reported favorable outcomes in terms of effectiveness and safety when neoadjuvant immunotherapy plus chemotherapy for operable locally advanced ESCC in the short term [31–36]. However, there is currently a lack of reports on the efficacy of immunotherapy plus chemotherapy in patients with unresectable locally advanced ESCC.

The combination of immunotherapy and chemoradiotherapy has emerged as a novel strategy for the treatment of EC, potentially exhibiting synergistic action and enhanced efficacy [37]. Although preliminary data from small-scale studies have demonstrated promising efficacy and reliable safety of immunotherapy combined with concurrent radiotherapy/chemoradiotherapy in unresectable locally advanced ESCC [38–42], there is still a lack of results from prospective phase III clinical studies to confirm the effectiveness of these combination treatments, leaving the optimal strategies unclear. At present, most of the clinical research on combination therapy in unresectable locally advanced ESCC focuses on concurrent administration of immunotherapy with CCRT or/and maintenance therapy after CCRT. However, no studies have reported on the use of induction immunotherapy plus chemotherapy prior to CCRT for this patient population. In this retrospective study, we aimed to evaluate the efficacy and safety profile of induction immunotherapy plus chemotherapy followed by definitive chemoradiotherapy in patients with unresectable locally advanced ESCC.

## Methods

### Patient selection

We retrospectively reviewed the medical records of patients with unresectable locally advanced ESCC who underwent radical CCRT at the First Affiliated Hospital of Sun Yat-sen University between January 2017 and December 2021. All eligible patients were aged between18 and 75 years, had histologically confirmed primary ESCC; presented with definitive endoscopic ultrasound and imaging evidence of cT1-4bN0/N + M0 (including inoperable, contraindications to surgery, or refusal of surgery) or exhibited M1 disease limited to supraclavicular lymph node metastases only. Additionally, patients included in this study demonstrated adequate hematologic, hepatic, and renal function. Patients with tumor bleeding, esophagus fistula, distant organ metastases, serious complications, severe active infections requiring systemic therapy, congenital or acquired immunodeficiencies, or psychiatric disorders were excluded from this retrospective study.

This study adhered to the principles outlined in the Declaration of Helsinki and was approved by the Ethics Committee of the First Affiliated Hospital of Sun Yat-sen University (LS [2022] No. 561).

### Treatments

A total of 132 patients with unresectable locally advanced ESCC who underwent radical CCRT were included in this study. The patients who received induction immunotherapy plus chemotherapy before CCRT were defined as the induction IC group, while those who did not receive such treatment were defined as the CCRT group.

In the induction IC group, during the phase of induction immunotherapy plus chemotherapy before CCRT, the anti-PD-1 immunotherapy regimen consisted of camrelizumab (Jiangsu Hengrui Medicine, China), sintilimab (Innovent Biologics, China), toripalimab (Shangha Merck & Co.), tislelizumab (BeiGene, China), or pembrolizumab (Merck & Co., USA). Additionally, dual-agent chemotherapy regimens including platinum agents (cisplatin or carboplatin) and taxane agents (paclitaxel or albumin paclitaxel) were administered.

The radiotherapy plans were delineated on localized computed tomography (CT) scan images with a resolution of 3–5 mm, obtained prior to the initiation of radiotherapy. The gross tumor volume (GTV) encompassed primary esophageal lesions (GTVp) and enlarged regional lymph nodes (GTVn), as identified through pre-treatment imaging and endoscopy. The clinical target volume (CTV) consisted of GTVp along with a superior and inferior expansion of 3 cm along the length of the esophagus, as well as a radial expansion of 1 cm; GTVn was expanded by 0.5–1.5 cm to include coverage of elective nodal regions. To generate the planning target volume (PTV), an expansion of 0.6–0.8 cm around both GTV and CTV was applied in all directions. A prescribed dose range between 45 and 54 Gy was administered to CTV, while GTV received a boost dose ranging from 50 and 66 Gy over a period of 5 to 6 weeks if organs at risk met dose constraints.

Concurrent chemotherapy included dual-agent or single-agent regimens based on platinum agent (cisplatin or carboplatin), taxane (paclitaxel or albumin paclitaxel or docetaxel), or pyrimidine (fluorouracil or capecitabine or S-1).

### Follow-up

Follow-up evaluations were conducted at 1 month post-treatment completion and subsequently every 3–6 months, encompassing medical history collection, physical examination, laboratory tests, and CT scans. Additionally, if clinically indicated, esophagogastroscopy, bronchoscopy, esophagography with barium or ultravist contrast agents, PET-CT scans, and other examinations were arranged. The last follow-up occurred on August 28, 2023. Independent radiologists evaluated the responses based on RECIST 1.1 criteria. Treatment-induced remission was categorized into complete response (CR), partial response (PR), stable disease (SD), and progressive disease (PD). The overall survival (OS) referred to the time from treatment initiation until death from any cause, and progression-free survival (PFS) denoted the time from treatment initiation until first documented disease progression or death from any cause. Toxicity assessment followed Common Terminology Criteria For Adverse Events Version 5.0 (CTCAE 5.0).

### Statistical analysis

Statistical analysis was conducted using Statistical Product and Service Solutions (SPSS) 27.0 software. The baseline characteristics and treatment relevance of the case data in both groups were compared using independent sample t test, chi-square test and Fisher’s exact test. Propensity score matching (PSM) analysis was employed to minimize selection bias between the two groups and ensure balanced patient characteristics. Patients in the induction IC group and CCRT group were matched with a 1:1 propensity score according to gender, age, history of tobacco and alcohol use, tumor location, tumor length, disease stage, etc. Prognostic factors potentially associated with PFS and OS were analyzed using Kaplan–Meier survival analysis, Log-Rank test, COX proportional risk regression models among others. All reported P values were two-sided with significance levels set to *P* < 0.05. *R* version 4.0 was utilized for generating survival curve plots.

## Results

### Patient characteristics

Among the 132 patients included in this study, 61 (45.26%) were assigned to the induction IC group, while 71 (54.74%) were allocated to the CCRT group. The baseline characteristics of the patients in both groups were shown in Table 1, and there were no significant differences observed in terms of age, gender, smoking history, pre-treatment weight loss, Karnofsky Performance Status Scale (KPS) score, tumor length, stage and reasons for not having surgery (*P* > 0.05). The majority of patients were male with predominantly T3 and T4 stages. Most patients (91.67%) had stage III and IV disease at presentation, a small number of patients (8.33%) with relatively early esophageal cancer who were at high risk for surgery due to advanced age or cardiopulmonary disease, or cervical esophageal cancer, or who refused surgery. The proportion of patients with only supraclavicular lymph node metastasis was similar between the two groups at approximately 18.03 and 21.13%, respectively. To further minimize confounding factors' influence on our study results, a PSM analysis was conducted resulting in a total of 50 pairs (100 patients) being enrolled for analysis purposes; no statistically significant differences were found regarding baseline data among these matched patient pairs (*P* > 0.05). Prior to PSM procedure implementation, there existed a discrepancy between the two groups concerning alcohol history; however, after matching was performed this difference became non-significant.

**Table 1:** Patient demographics and baseline characteristics

Characteristics	Before propensity score matching, *n* (%) (*n* = 132)			After propensity score matching, *n* (%) (*n* = 100)		
	Induction IC group	CCRT group	*P* value	Induction IC group	CCRT group	*P* value
	(*n* = 61)	(*n* = 71)		(*n* = 50)	(*n* = 50)	
Gender			*0.551*			*1.000*
Male	52(88.73)	63(85.25)		43(86.00)	43(86.00)	
Female	9(11.27)	8(14.75)		7(14.00)	7(14.00)	
Age (years)			*0.126*	60.5 (41–74)	62 (43–75)	*0.829*
< 65	38(62.30)	53(74.65)		34(68.00)	35(70.00)	
≥ 65	23(37.70)	18(25.35)		16(32.00)	15(30.00)	
Smoking status			*0.055*			*0.834*
Former or current	35(57.38)	52(73.24)		32(64.00)	33(66.00)	
Never	26(42.62)	19(26.76)		18(36.00)	17(34.00)	
Alcohol consumption			***0.032***			*0.840*
Former or current	30(49.18)	48(67.61)		28(56.00)	29(58.00)	
Never	31(50.82)	23(32.39)		22(44.00)	21(42.00)	
Weight loss before treatment			*0.345*			*0.822*
≤ 5%	45(73.77)	47(66.20)		37(74.00)	36(72.00)	
> 5%	16(26.23)	24(33.80)		13(26.00)	14(38.00)	
KPS			*0.989*			*0.841*
< 90	31(50.82)	36(50.70)		25(50.00)	24(48.00)	
≥ 90	30(49.18)	35(49.30)		25(50.00)	26(52.00)	
Tumor length (cm)	5.70 ± 2.39	6.19 ± 3.07	*0.314*	5.95 ± 2.29	5.80 ± 2.48	*0.754*
Tumor location			*0.822*			*0.529*
Upper segment	19(31.15)	22(30.99)		19(38.00)	14(28.00)	
Middle segment	19(31.15)	26(36.62)		13(26.00)	19(38.00)	
Lower segment	21(34.42)	22(30.99)		16(32.00)	16(32.00)	
Multipart	2(3.28)	1(1.40)		2(4.00)	1(2.00)	
T stage			*0.309*			*0.610*
T1	0(0.00)	1(1.41)		0(0.00)	1(2.00)	
T2	7(11.47)	7(9.86)		5(10.00)	6(12.00)	
T3	33(54.10)	29(40.84)		28(56.00)	23(46.00)	
T4	21(34.43)	34(47.89)		17(34.00)	20(40.00)	
N stage			*0.306*			*0.653*
N0	4(6.56)	7(9.86)		4(8.00)	2(4.00)	
N1	17(27.87)	27(38.03)		15(30.00)	18(36.00)	
N2	27(44.26)	29(40.84)		22(44.00)	24(48.00)	
N3	13(21.31)	8(11.27)		9(18.00)	6(12.00)	
M stage			*0.656*			*0.424*
M0	50(81.97)	56(78.87)		40(80.00)	43(86.00)	
M1	11(18.03)	15(21.13)		10(20.00)	7(14.00)	
Tumor stage			*0.687*			*0.713*
I	0(0.00)	1(1.41)		0(0.00)	1(2.00)	
II	4(6.56)	6(8.45)		4(8.00)	4(8.00)	
III	27(44.26)	24(33.80)		22(44.00)	20(40.00)	
IVA	19(31.15)	25(35.21)		14(28.00)	18(36.00)	
IVB	11(18.03)	15(21.13)		10(20.00)	7(14.00)	
Reasons for not having surgery			*0.419*			*0.603*
Unresectable tumors	56(91.80)	66(92.96)		46(92.00)	45(90.00)	
Contraindications to surgery	2(3.28)	4(5.63)		2(4.00)	4(8.00)	
Refusal of surgery	3(4.92)	1(1.41)		2(4.00)	1(2.00)	

*IC* Induction immunotherapy plus chemotherapy; *CCRT* Concurrent chemoradiotherapy; *KPS* Karnofsky performance status scale

### Assessment of efficacy

The induction IC group exhibited a significantly higher overall objective response rate (ORR) compared to the CCRT group (85.24 vs. 26.76%, *P* < 0.001) (Table 2). In the induction IC group, CR was achieved by 9 patients (14.75%) and PR was achieved by 43 patients (70.49%). Furthermore, following administration of induction immunotherapy plus chemotherapy, CR was observed in 2 patients (3.28%) and PR in 48 patients (78.69%).

**Table 2:** Evaluation of efficacy after treatment

Tumor response	Induction IC Group			CCRT group
	After induction therapy, *n* (%)	After CCRT, *n* (%)	After all treatments, *n* (%)	After CCRT, *n* (%)
CR	2 (3.28)	5 (8.20)	9 (14.75)	1 (1.41)
PR	48 (78.69)	17 (27.87)	43 (70.49)	18 (25.35)
SD	9 (14.75)	36 (59.01)	6 (9.84)	44 (61.97)
PD	2 (3.28)	1 (1.64)	1 (1.64)	5 (7.04)
NE	0 (0.00)	2 (3. 28)	2 (3.28)	3 (4.23)

*IC* Induction immunotherapy plus chemotherapy; *CCRT* Concurrent chemoradiotherapy; *CR* Complete remission; *PR* Partial response; *SD* Stable disease; *SD* Progressive disease; *NE* Not evaluable

**Fig. 1:**
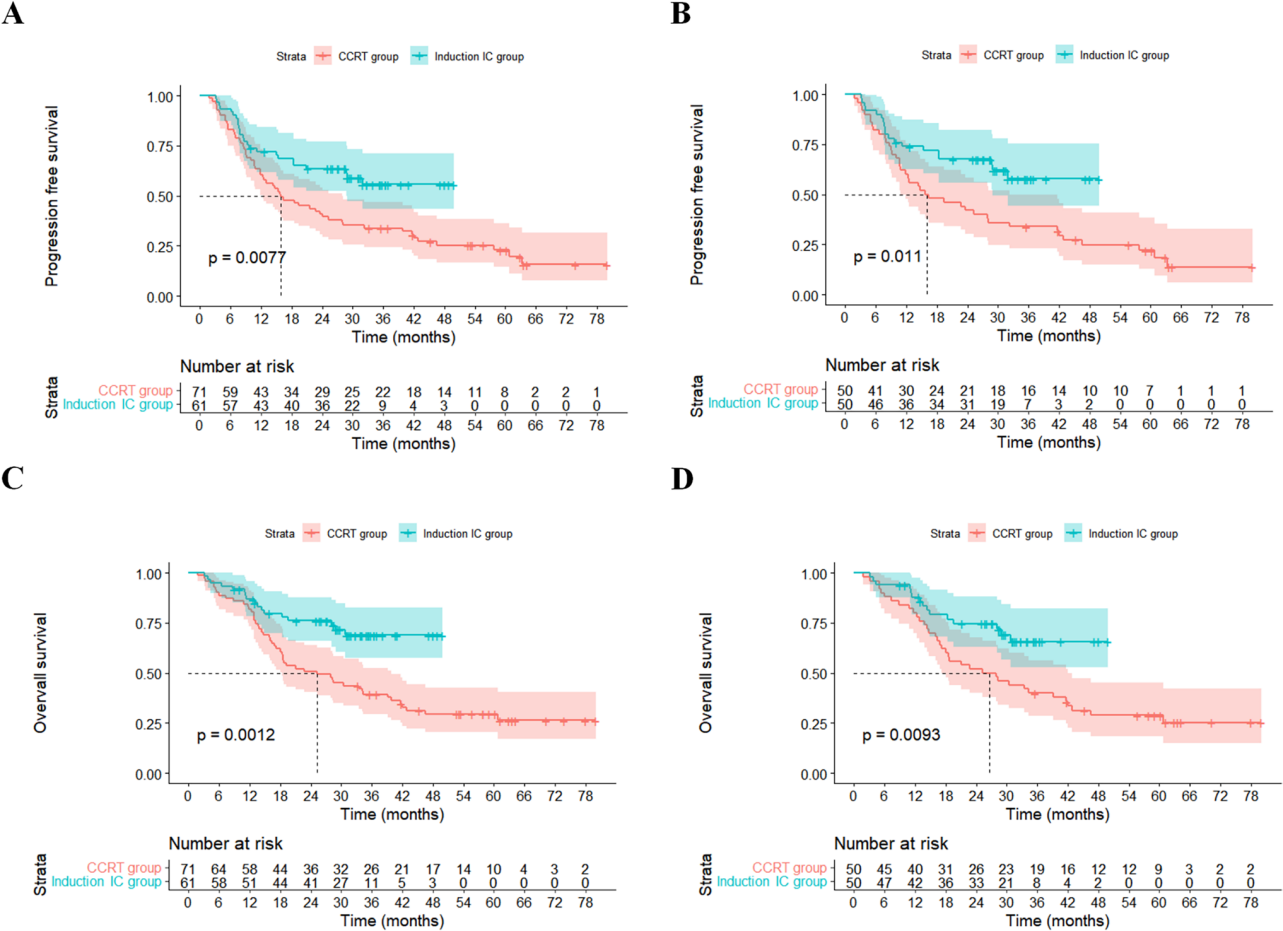
Progression-free survival (PFS) and Overall survival (OS) curves curves comparing patients in the induction immunotherapy plus chemotherapy group (induction IC group) with those in the concurrent chemoradiotherapy group (CCRT group). **A** Prior to propensity score matching (PSM), PFS was significantly different between patients in the induction IC group and CCRT group (*n* = 132, *P* = 0.0077); **B** After PSM, there remained a significant difference in PFS between patients in the induction IC group and CCRT group (*n* = 100, *P* = 0.011); **C** Before PSM, OS differed significantly between patients in the induction IC group and CCRT group (*n* = 132, *P* = 0.0012); **D** After PSM, OS still showed a significant difference between patients in the induction IC group and CCRT group (*n* = 100, *P* = 0.0093)

The median follow-up time for patients in this study was 37.0 months (95% confidence interval [CI] 32.0–42.1 months), and the median PFS was 25.2 months (95% CI 13.9–36.4 months). Among them, the induction IC group had an unreached median PFS, while the CCRT group had a median PFS of 15.9 months (hazard ratio [HR] 0.526, 95% CI 0.325–0.851, *P* = 0.0077). Patients in the induction IC group exhibited longer PFS compared to those in the CCRT group, with 1-year PFS rates of 72.1 and 60.6%, and 2-year PFS rates of 63.5 and 40.8%, respectively (Fig. 1A). After PSM, the median PFS for a cohort of 100 patients was 28.8 months (95% CI 13.5–44.2 months), and the median PFS in the induction IC group and the CCRT group was not reached (NR) and 15.6 months, respectively; indicating that after PSM, patients in the induction IC group demonstrated superior PFS compared to those in the CCRT group (HR 0.490, 95% CI 0.280–0.858, *P* = 0.011), with 1-year PFS rates of 73.9 and 60.0%, and 2-year PFS rates of 67.6 and 42.0%, respectively (Fig. 1B).

The median OS of 132 patients was 39.2 months (95% CI 29.5–48.9 months), with median OS not reached in the induction IC group, while in the CCRT group it was 25.2 months (HR 0.412, 95% CI 0.236–0.719, *P* = 0.0012). Patients in the induction IC group had significantly longer OS compared to those in the CCRT group, with 1-year OS rates of 86.7 and 81.7%, and 2-year OS rates of 76.0 and 50.7%, respectively (Fig. 1C). After PSM, the median OS of 100 patients was 39.2 months (95% CI 27.9–50.4 months), and the median OS in the induction IC group and the CCRT group were NR and 25.2 months (HR 0.454, 95% CI 0.246–0.837, *P* = 0.0093), respectively. After PSM, patients in the induction IC group had longer OS than those in the CCRT group, with 1-year OS rates of 87.7 and 80.0%, and 2-year OS rates of 74.6 and 52.0%, respectively (Fig. 1D).

### Prognostic factors

Prognostic factors were assessed through univariate and multivariate COX analysis, including age, sex, smoking history, alcohol history, pre-treatment weight loss, KPS score, number of primary tumors, tumor length, TNM stage of tumor, radiotherapy dose, concurrent chemotherapy use and the administration of induction immunotherapy plus chemotherapy. The results demonstrated T stage (HR 2.295, 95% CI 1.441–3.656, *P* < 0.001), N stage (HR 7.239, 95% CI 1.741–30.100, *P* = 0.006), regimen of concurrent chemotherapy (HR 3.245, 95% CI 1.399–7.525, *P* = 0.006), and the utilization of induction immunotherapy plus chemotherapy (HR 0.564, 95%CI 0.345–0.920, *P* = 0.022) were identified as independent prognostic factors for PFS (Table 3).

**Table 3:** Prognostic factors of PFS by univariate and multivariate analysis

Variables	Univariate analysis		Multivariate analysis	
	HR (95%CI)	*P* value	HR (95%CI)	*P* value
Gender (Female vs. Male)	1.458 (0.699–3.041)	0.315		
Age (< 65 vs. ≥ 65)	0.828 (0.506–1.357)	0.455		
Smoking status (Never vs. Former or current)	1.231 (0.758–1.999)	0.401		
Alcohol consumption (Never vs. Former or current)	1.779 (1.107–2.859)	0.017	1.162 (0.706–1.913)	0.554
Weight loss (≤ 5% vs. > 5%)	1.450 (0.906–2.320)	0.121		
KPS (< 90 vs. ≥ 90)	0.660 (0.424–1.027)	0.065		
Number of primary tumors (Single vs. Multiple)	1.506 (0.368–6.156)	0.569		
Tumor length	1.067 (0.996–1.142)	0.064		
T stage (T1-3 vs. T4)	2.389 (1.525–3.743)	< 0.001	2.295 (1.441–3.656)	< 0.001
N stage (N0 vs. N +)	5.850 (1.430–23.927)	0.014	7.239 (1.741–30.100)	0.006
M stage (M0 vs. M1)	1.108 (0.654–1.877)	0.703		
Radiotherapy dose (Gy) (≤ 60.2 vs. > 60.2)	1.085 (0.699–1.684)	0.716		
Regimen of concurrent chemotherapy (Single agent vs. Double agents)	2.911 (1.264–6.701)	0.012	3.245 (1.399–7.525)	0.006
Whether induced immunotherapy plus chemotherapy was received (No vs. Yes)	0.526 (0.325–0.851)	0.009	0.564 (0.345–0.920)	0.022

*PFS* Progression-free survival; *HR* Hazard ratio; *CI* Confidential interval; *VS* Versus; *KPS* Karnofsky performance status scale

Additionally, univariate and multivariate COX analysis was conducted to evaluate potential prognostic factors of OS. The findings revealed that *N* stage (HR 4.476, 95%CI 1.075–18.640, *P* = 0.040), regimen of concurrent chemotherapy (HR 2.960, 95%CI 1.176–7.451, *P* = 0.021), and the administration of induction immunotherapy plus chemotherapy was received (HR 0.443, 95% CI 0.253–0.777, *P* = 0.005) were considered as significant independent prognostic factors for OS (Table 4).

**Table 4:** Prognostic factors of OS by univariate and multivariate analysis

Variables	Univariate analysis		Multivariate analysis	
	HR (95%CI)	*P* value	HR (95%CI)	*P* value
Gender (Female vs. Male)	1.895 (0.761–4.719)	0.169		
Age (< 65 vs. ≥ 65)	1.033 (0.611–1.748)	0.903		
Smoking status (Never vs. Former or current)	1.206 (0.708–2.053)	0.490		
Alcohol consumption (Never vs. Former or current)	1.865 (1.105–3.148)	0.020	1.351 (0.772–2.365)	0.293
Weight loss (≤ 5% vs. > 5%)	1.769 (1.070–2.926)	0.026	1.325 (0.757–2.321)	0.325
KPS (< 90 vs. ≥ 90)	0.588 (0.362–0.954)	0.032	0.652 (0.381–1.117)	0.120
Number of primary tumors (Single vs. Multiple)	1.800 (0.439–7.381)	0.414		
Tumor length	1.097 (1.026–1.172)	0.006	0.991 (0.918–1.070)	0.820
T stage (T1-3 vs. T4)	2.218 (1.367–3.601)	0.001	1.650 (0.956–2.847)	0.072
N stage (N0 vs. N +)	4.284 (1.047–17.538)	0.043	4.476 (1.075–18.640)	0.040
M stage (M0 vs. M1)	1.124 (0.632–1.998)	0.691		
Radiotherapy dose (Gy) (≤ 60.2 vs. > 60.2)	1.224 (0.756–1.982)	0.411		
Regimen of concurrent chemotherapy (Single agent vs. Double agents)	2.717 (1.091–6.764)	0.032	2.960 (1.176–7.451)	0.021
Whether induced immunotherapy plus chemotherapy was received (No vs. Yes)	0.412 (0.236–0.719)	0.002	0.443 (0.253–0.777)	0.005

*OS*: Overall survival; *HR* Hazard ratio; *CI* Confidential interval; *VS* Versus; *KPS* Karnofsky performance status scale

### Adverse events

The most frequent treatment-related adverse events (TRAEs) of any grade were myelosuppression and radiation esophagitis (Table 5). The incidence of grade 3 or higher TRAEs in the in the induction IC group and CCRT group was 44.26 and 54.93%, respectively. Significantly lower incidences of grade 3 or worse leukopenia (18.30% vs. 40.85%, *P* = 0.004) and neutropenia (8.20 vs. 26.76%, *P* = 0.006) were observed in the induction IC group compared to the CCRT group, with one patient from each group dying due to septic shock secondary to myelosuppression after CCRT administration. The incidence rates of grade 3 or worse alanine aminotransferase elevation and aspartate aminotransferase elevation in the IC group were significantly higher than those in the CCRT group, with values of 9.84 and 1.41% (*P* = 0.048), 8.20 and 0.00% (*P* = 0.019), respectively. The incidences rates of grade 3 or worse thrombocytopenia, anemia, and radiation esophagitis were comparable between both groups. The most common immune-related adverse events in the induction IC group included grade 1 or 2 rash (7/61, 11.48%), pruritus (10/61, 16.39%), interstitial pneumonia (1/61, 1.64%), and hypothyroidism (3/61, 4.92%).

**Table 5:** Treatment-related adverse events

	Any grade, *n* (%)			Grade ≥ 3, n (%)		
	Induction IC group	CCRT group	*P* value	Induction IC group	CCRT group	*P* value
Leukopenia	45 (73.77)	54 (76.06)	*0.762*	11 (18.30)	29 (40.85)	**0.004**
Neutropenia	32 (52.46)	38 (53.52)	*0.903*	5 (8.20)	19 (26.76)	**0.006**
Thrombocytopenia	35 (57.38)	32 (45.07)	*0.159*	4 (6.56)	4 (5.63)	1.000
Anemia	61 (100.00)	65 (91.55)	***0.030***	16 (26.23)	18 (25.35)	0.909
Elevated alanine aminotransferase	28 (45.90)	18 (25.35)	***0.013***	6 (9.84)	1 (1.41)	**0.048**
Elevated aspartate aminotransferase	32 (52.46)	10 (14.08)	** < *0.001***	5 (8.20)	0 (0.00)	**0.019**
Elevated serum creatinine	6 (9.84)	7 (9.86)	*0.996*	0 (0.00)	1 (1.41)	1.000
Nausea	37 (60.66)	29 (40.85)	***0.023***	0 (0.00)	1 (1.41)	1.000
Vomiting	29 (47.54)	15 (21.13)	***0.001***	0 (0.00)	0 (0.00)	–
Diarrhea	8 (13.11)	6 (8.45)	*0.386*	0 (0.00)	1 (1.41)	1.000
Fatigue	7 (11.48)	10 (14.08)	*0.655*	0 (0.00)	0 (0.00)	–
Radiation esophagitis	37 (60.66)	56 (78.87)	***0.022***	4 (6.56)	4 (5.63)	1.000
Radiation pneumonitis	5 (8.20)	4 (5.63)	*0.732*	0 (0.00)	1 (1.41)	1.000
Rash	7 (11.48)	0 (0.00)	***0.004***	0 (0.00)	0 (0.00)	–
Pruritus	10 (16.39)	0 (0.00)	** < *0.001***	0 (0.00)	0 (0.00)	–
Interstitial pneumonia	1 (1.64)	0 (0.00)	*0.462*	0 (0.00)	0 (0.00)	–
Hypothyroidism	3 (4.92)	0 (0.00)	*0.096*	0 (0.00)	0 (0.00)	–

*IC* Induction immunotherapy plus chemotherapy; *CCRT* Concurrent chemoradiotherapy

## Discussion

To the best of our knowledge, this study represents the first investigation comparing the efficacy and safety of induction immunotherapy in combination with chemotherapy followed by concurrent chemoradiotherapy for managing unresectable locally advanced ESCC. Our findings demonstrate a significant improvement in OS among patients who received induction immunotherapy plus chemotherapy compared to those who underwent definitive concurrent chemoradiotherapy alone, with 1-year OS rates of 86.7 and 81.7%, and 2-year OS rates of 76.0 and 50.7%, respectively. This retrospective analysis provides novel evidence supporting the potential adoption of an alternative treatment paradigm involving induction immunotherapy plus chemotherapy followed by concurrent chemoradiotherapy for patients with unresectable locally advanced ESCC.

In the pre-immunotherapy era, the efficacy of induction chemotherapy in improving survival outcomes for patients with unresectable locally advanced ESCC was a subject of controversy based on existing research findings [17–20]. A randomized phase II trial demonstrated no significant improvement in response rate or survival when induction chemotherapy was added to CCRT in unselected ESCC patients [20]. One possible reason for the failure of this study could be attributed to the limited effectiveness of the induction chemotherapy regimen. Interestingly, post-hoc analysis revealed that responders to induction chemotherapy had significantly better survival compared to nonresponders among EC patients treated with CCRT. In the current era of immunotherapy, combination therapy with immunotherapy and chemotherapy has shown ORR as high as 70% in advanced ESCC cases [21, 22, 25, 29, 30]. Furthermore, neoadjuvant immunotherapy plus chemotherapy in operable ESCC cases resulted in a pooled pathological complete response (pCR) rate of 32.4% and major pathological response (MPR) rate of 49.4% [43]. Our findings demonstrated that induction immunotherapy plus chemotherapy achieved CR or PR in a remarkable proportion of patients within the induction IC group (81.97%), highlighting its substantial role within the comprehensive treatment plan. This is likely one key factor contributing to the significant survival benefit observed in our study.

Furthermore, tumors exhibited shrinkage following induction immunotherapy plus chemotherapy, thereby rendering them more susceptible to radiation therapy due to an enhanced tumor microenvironment characterized by reduced hypoxia and promoted normalization of tumor blood vessels, along with increased infiltration of T cells into the tumor tissues [44]. Consequently, the sequential application of immunotherapy plus chemotherapy has resulted in improved sensitivity toward radiotherapy. This represents a second potential explanation for the observed efficacy of concurrent chemoradiotherapy following prior administration of immunotherapy plus chemotherapy in our current study.

The combination of immunotherapy with CCRT holds significant potential for future applications in the management of unresectable locally advanced ESCC. Various modalities exist for combining immunotherapy with radiotherapy to treat esophageal cancer, including pre-radiotherapy immunotherapy, concurrent radiotherapy immunotherapy, post-radiotherapy immunotherapy, or different combinations thereof. Radiotherapy plays a crucial role in releasing tumor antigens and modulating immune pathways favorably, thereby enhancing tumor antigen presentation, priming of tumor-specific cytotoxic T cells, as well as improving T cell homing, engraftment and function within tumors, which may improve the response of the tumor to immunotherapy [45]. Consequently, current clinical research on combination therapy in unresectable locally advanced ESCC predominantly focuses on CCRT combined with concomitant or/and adjuvant immunotherapy [38–42].

Several studies have suggested that the combination of CCRT with concomitant or/and adjuvant immunotherapy extended the survival benefit for patients with unresectable locally advanced ESCC. Park et al.'s investigation demonstrated impressive 2-year OS rates of 75% among individuals with locally advanced ESCC when durvalumab and tremelimumab were combined with definitive chemoradiotherapy [38]. Similarly, another phase Ib clinical trial evaluating camrelizumab combined with concurrent chemoradiotherapy reported high 1-year and 2-year OS rates of patients up to 85.0 and 69.6%, respectively [39]. On the other hand, Zhu et al.’s results revealed that more than 60% of patients achieved clinical CR rates when treated with toripalimab combined with concurrent chemoradiotherapy; the corresponding 1-year OS rate was 78.4%, while 1-year PFS was 54.5% [42]. Although preliminary data from these small-scale studies have shown promising efficacy and safety profiles of CCRT in combination with concomitant or/and adjuvant immunotherapy in unresectable locally advanced ESCC [38–42, 46], there is still a lack of results from prospective phase III clinical trials (such as ESCORT-CRT, KEYNOTE-975, KUNLUN, RATIONALE-311, KYSCRAPER-07) [47–50] to confirm the effectiveness of these combination treatments, leaving the optimal strategies unclear. Notably, our retrospective study's 2-year OS rate was comparable or even superior to those observed in these previous small-scale prospective studies; this further emphasizes the need for additional clinical trials investigating the efficacy of induction immunotherapy plus chemotherapy followed by CCRT. 

There were some differences observed in treatment-related adverse events between the two groups in this study. Notably, patients in the induction IC group exhibited a lower incidence of adverse events such as leukopenia, neutropenia, nausea, and vomiting. This favorable outcome could potentially be attributed to the utilization of long-acting recombinant human granulocyte colony-stimulating factor and novel anti-emetic drugs like neurokinin-1 (NK-1) receptor antagonists that have emerged in recent years. Conversely, patients in the induction IC group experienced a higher incidence of hepatic dysfunction; however, most cases were classified as grade 1–2 and showed improvement with liver protective agents. No significant disparities were observed regarding radiation pneumonitis between the two groups, suggesting that induction immunotherapy plus chemotherapy may not exacerbate radiation-induced lung inflammation. Overall, the treatment approach involving sequential concurrent chemoradiotherapy following induction immunotherapy plus chemotherapy demonstrates acceptability.

As a retrospective, single-center study, our investigation was limited by a small sample size and selection bias; therefore, further prospective, randomized controlled clinical trials are warranted to elucidate the efficacy of this treatment model. Moreover, the follow-up duration in our study remained relatively short, with median PFS and OS not yet reached in the induction IC group. Consequently, an extended follow-up period is necessary to ascertain the long-term effectiveness of this combination therapy approach. Additionally, due to immunotherapy being a novel anti-tumor treatment modality introduced later on, there exists a notable disparity in treatment timing between the two patient groups included in our study. Notably, patients in the induction IC group initiated treatment slightly later than those receiving CCRT. The potential impact of this factor on patient prognosis remains unclear; hence, more prospective randomized controlled studies are imperative. Furthermore, as a retrospective study, there were differences in the immune checkpoint inhibitors and chemotherapy drugs selected by patients in treatment, which may also affect the efficacy, and more prospective studies are needed to clarify the impact of different drugs on efficacy.

## Conclusions

In this retrospective study, we first have demonstrated the efficacy and safety of induction immunotherapy plus chemotherapy followed by concurrent chemoradiotherapy for the management of unresectable locally advanced ESCC. These findings highlight the potential of this novel treatment approach, emphasizing the need for further investigation through prospective clinical trials.

## Data Availability

Data can be made available upon reasonable request.
